# General lifestyle interventions on their own seem insufficient to improve the level of physical activity after stroke or TIA: a systematic review

**DOI:** 10.1186/s12883-020-01730-3

**Published:** 2020-05-01

**Authors:** Wendy Hendrickx, Lara Vlietstra, Karin Valkenet, Roderick Wondergem, Cindy Veenhof, Coralie English, Martijn Frits Pisters

**Affiliations:** 1 Department of Rehabilitation, Physiotherapy Science & Sport, UMC Utrecht Brain Center, University Medical Center Utrecht, Utrecht University, Utrecht, The Netherlands; 2 Center for Physical Therapy Research and Innovation in Primary Care, Julius Health Care Centers, Emile Hullebroeckstraat 60, 3543 BZ Utrecht, The Netherlands; 3 Department of Medicine, Dunedin School of Medicine and School of Physiotherapy, University of Otago, Dunedin, New Zealand; 4 Department of Health Innovations and Technology, Fontys University of Applied Sciences, Eindhoven, The Netherlands; 5 Innovation for Human Movement Care Research Group, HU University of Applied Sciences, Utrecht, The Netherlands; 6 School of Health Sciences and Priority Research Centre for Stroke and Brain Injury, University of Newcastle, Newcastle, Australia; 7 Centre for Research Excellence in Stroke Recovery and Rehabilitation, Florey Institute of Neuroscience and Hunter Medical Research Institute, Newcastle, Australia

**Keywords:** Stroke, Lifestyle, Risk reduction behaviour, Secondary prevention, Exercise

## Abstract

**Background:**

Insufficient amounts of physical activity is a risk factor for (recurrent) stroke. People with a stroke or transient ischemic attack (TIA) have a high risk of recurrent stroke and have lower levels of physical activity than their healthy peers. Though several reviews have looked at the effects of lifestyle interventions on a number of risk factors of recurrent stroke, the effectiveness of these interventions to increase the amounts of physical activity performed by people with stroke or TIA are still unclear. Therefore, the research question of this study was: what is the effect of lifestyle interventions on the level of physical activity performed by people with stroke or TIA?

**Method:**

A systematic review was conducted following the guidelines of the Preferred Reporting Items for Systematic Reviews and Meta-analyses (PRISMA) statement. Pubmed, Embase and Cumulative Index for Nursing and Allied Health Literature (CINAHL), were searched up to August 2018. Randomised controlled trials that compared lifestyle interventions, aimed to increase the amount of physical activity completed by participants with a stroke or TIA, with controls were included. The Physiotherapy Evidence Database (PEDro) score was used to assess the quality of the articles, and the Grading of Recommendations, Assessment, Development and Evaluations (GRADE) method for the best evidence synthesis.

**Results:**

Eleven trials (*n* = 2403) met the inclusion criteria. The quality of the trials was mostly high, with 8 (73%) of trials scoring ≥6 on the PEDro scale. The overall best evidence syntheses showed moderate quality evidence that lifestyle interventions do not lead to significant improvements in the physical activity level of people with stroke or TIA. There is low quality evidence that lifestyle interventions that specifically target physical activity are effective at improving the levels of physical activity of people with stroke or TIA.

**Conclusion:**

Based on the results of this review, general lifestyle interventions on their own seem insufficient in improving physical activity levels after stroke or TIA. Lifestyle interventions that specifically encourage increasing physical activity may be more effective. Further properly powered trials using objective physical activity measures are needed to determine the effectiveness of such interventions.

**Trial registration:**

PROSPERO, CRD42018094437.

## Background

Cardiovascular disease is the leading cause of death and disability globally [1]. Cerebrovascular diseases, including stroke and transient ischemic attack (TIA), account for 34% of cardiovascular disease in males and 37% in females [1]. This equates to approximately 15 million people worldwide having a stroke or TIA each year [1]. Due to improvements in acute stroke treatment, survival rates are improving in several parts of the world [1]. However, people who have had a stroke or TIA are at high risk (40% in 10 years) of having a recurrent stroke [2, 3]. Therefore, secondary prevention is vital.

Insufficient levels of physical activity is one of the strongest modifiable risk factors of stroke and recurrent stroke [1, 4, 5]. The World Health Organisation, the American Heart Association and the American Stroke Association recommend 150 min per week of moderate-intensity aerobic activity or 75 min per week of vigorous aerobic activity, or a combination of both, preferably spread throughout the week and preferably performed in bouts of at least 10 min duration [6–8]. However, recent studies have shown that the levels of physical activity performed by people with a stroke or TIA do not meet these recommendations and are low compared to the physical activity levels of healthy peers [9–11]. Thus, it appears that people with stroke and TIA require additional interventions to support them to improve their level of physical activity.

Several multimodal lifestyle interventions have been developed, incorporating educational, motivational and other psychosocial components with the aim to support behaviour change to reduce risk factors of recurrent stroke, including improving physical activity levels for people after stroke or TIA. Since improving physical activity is recommended in Stroke Clinical Guidelines internationally [12–15], it is important to know if these lifestyle interventions are effective in order to guide clinical practice. Three earlier similar reviews have been conducted. The first review only included trials published up to 2009 [16], and found insufficient evidence to determine the effects of lifestyle interventions on the levels of physical activity. The second review was also inconclusive [17], both recommend further high quality research [16, 17]. The most recent review [18], including trials published up to May 2015, concluded that a meta-analyses on physical activity was not possible due to diversity in the outcome measures used [18]. A best evidence synthesis including comparison of the intervention effect to controls and weighing the quality of the included trials was not conducted nor was an effect estimate of the interventions provided [18]. It remains unclear if lifestyle interventions are effective in improving the levels of physical activity performed by people with stroke or TIA. Furthermore, the need to include strategies that specifically focus on the levels of physical activity, e.g. supervised exercise, is unclear. A review specifically examining the effects of lifestyle interventions on physical activity after stroke is needed to support physiotherapists’ clinical practice. Therefore, the research question for this systematic review was: What is the effect of lifestyle interventions on the level of physical activity performed by people with stroke or TIA?

## Methods

This systematic review was conducted in accordance with the guidelines of the Preferred Reporting Items for Systematic Reviews and Meta-Analyses (PRISMA) statement [19], and is registered with the International Prospective Register of Systematic Reviews (PROSPERO; CRD42018094437).

### Eligibility criteria

Trials were eligible for inclusion if:

1. the participants were adults with clinically confirmed stroke or TIA;
2. the intervention was a lifestyle or behavioural intervention, defined as an intervention that incorporated educational, motivational and other psychosocial components with the aim to support behaviour change to reduce risk factors of recurrent stroke;
3. the study design was a randomised clinical trial (RCT) where the lifestyle intervention was compared with ‘no intervention’, ‘placebo’ and/or ‘usual care’;
4. at least one outcome measure of physical activity (any form of light physical activity and/or moderate to vigorous physical activity) was reported;
5. the full text article was available in English or Dutch.

Trials defined in the manuscript as a pilot or feasibility trial were excluded because of likely insufficient power to show effect.

### Search

Three electronic databases, Pubmed, Embase and CINAHL, were searched up to August 2018. The search strategy was constructed in Pubmed and adapted for CINAHL and Embase, see supplementary Additional file 1 (‘Search Strategy’) for the search strategy. We also scanned reference lists of relevant previous reviews identified in the initial orientation search and in the systematic search, for any additional relevant citations [16–18].

### Study selection

All trials identified in the search were first screened by title and abstract, then full-texts reviewed to determine eligibility. The study selection was independently conducted by the 2 authors (WH and LV). Disagreements were resolved by discussion. If no consensus could be reached, a third author (MFP) was consulted.

### Data extraction

Data extraction included descriptive data, demographics of study populations, sample sizes, the content of the intervention and the control, duration of the intervention, outcome measures on physical activity, time points of measurement and the study results. Data were extracted by one author (WH) and checked by a second author (LV) with disagreements resolved by discussion. If no consensus could be reached a third author (MFP) was consulted.

### Quality appraisal

The PEDro scale for RCTs and controlled clinical trials was used to determine the methodological quality of the included trials [20]. The PEDro scale consists of 11 ‘yes’ or ‘no’ statements with regards to domains like randomisation, blinding, attrition and reporting of results (see supplementary Table S1, PEDro scale). Points are only awarded when a criterion is clearly satisfied [20]. The highest possible score is 10 points (item 1 is not scored) [20]. Trials with a total score of 6 or higher are considered to be of high quality [21]. The quality appraisal was independently completed by 2 authors (WH and LV). The results were compared to see if there were any differences. If so, these were discussed. If no consensus could be reached a third author (MFP) was consulted.

### Best evidence synthesis

A meta-analysis was the preferred synthesis method. However, due to heterogeneity of outcome measures in the different trials, this was not possible. Instead, a best evidence synthesis was conducted, based on the available results from the included trials. We used the best evidence synthesis method from the Grading of Recommendations, Assessment, Development and Evaluations (GRADE) Working Group [22–25]. This method combines the consistency of the findings with the quality of the included trials. The domains for high quality evidence are [22–25]:

1. At least 75% of the RCTs with no limitations of study design have consistent findings,
2. Direct data, (this refers generalisability, the extent to which the people, interventions and outcomes in the trials are comparable to those defined in the inclusion criteria of the review).
3. Precise data, (this refers to a sufficient number of participants and events and the width of the confidence intervals).
4. No known or suspected publication biases.

For each domain for ‘high quality evidence’, that is not met, the level of evidence is downgraded [22–25]:

- High quality evidence: At least 75% of the RCTs with no limitations of study design have consistent findings, direct and precise data and no known or suspected publication biases;
- Moderate quality evidence: 1 of the above domains is not met;
- Low quality evidence: 2 of the above domains are not met;
- Very low quality evidence: 3 of the above domains are not met.

### Effect size of the intervention and subgroup analyses

To determine the effect size of the interventions, the standardized mean difference (SMD), including the 95% confidence intervals, was calculated where possible for the between group differences at follow-up [26]. A SMD of ≥0.2 was considered a small effect, ≥0.5 a moderate effect, and ≥ 0.8 a large effect of exercise therapy as stated by Cohen et al. [27]. Subgroup analyses were performed based on the content of the intervention, i.e. the inclusion of specific strategies targeting improving the level of physical activity in people with stroke or TIA.

## Results

### Flow of trials through the review

**Fig. 1:**
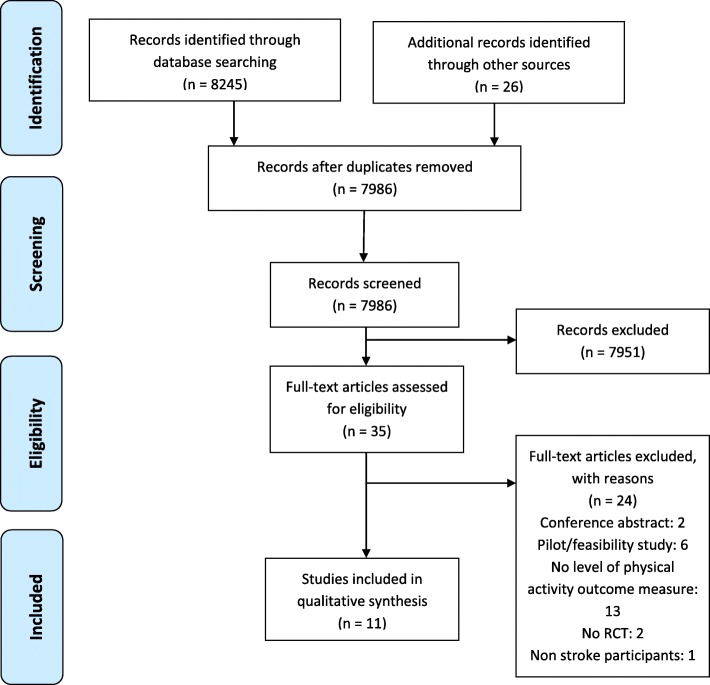
PRISMA Flow diagram

A total of 8245 articles were identified in the literature search. When duplicates were removed, 7986 articles remained. After screening the titles and abstracts, 35 articles progressed to full text review, of which 11 trials were included (Fig. 1, ‘PRISMA Flow diagram’).

### Characteristics of participants and trials

Characteristics of included trials are reported in Table 1, ‘Summary of included trials’. The 11 included trials reported data from *n* = 2403 participants (*n* = 1205 intervention and 1198 control). The mean age ranged from 57 to 72 years. In all trials, stroke or TIA was clinically diagnosed in a hospital [28–38], and most had a mild stroke or TIA [28–31, 33–37], and enrolled in the trials after returning home [29–38]. There was a wide range in the sample sizes, ranging from 29 to 283 per trial arm. Most trials (73%) targeted multiple risk factors without a specific focus on improving the levels of physical activity [29–33, 35, 37, 38]. Three trials (27%) specifically targeted improving physical activity [28, 34, 36].

**Table 1:** Summary of included trials

Study	Participants Age, *yr* mean (SD or range) or median (IQR, IQR) Sex, *n male*	Intervention		Frequency and duration of interventions	Outcome measures
		Content	Discipline delivering the intervention mode of delivery		
Kono et al. [28] (2013)	Time since stroke unknown Exp: *n* = 35 Age 64 (7) Sex 21 M Con: *n* = 35 Age 63 (11) Sex 27 M	Exp: physical activity coaching + supervised exercise + home exercise program + a salt intake reduction e-learning program Con: 3 sessions advice to facilitate healthy lifestyle	Exp: health care professional interventionist, physiotherapist Face-to-face Con: health care professional interventionist Face-to-face	Exp: 3/wk. for 24 wk. Con: 3 sessions in 24 wk	Steps/day (accelerometer) Time in high, moderate and light intensity physical activity min/day (accelerometer)
Gillham et al. [29] (2010)	Time since stroke or TIA unknown Exp: *n* = 26 Age 68 (12) Sex 4 M Con: *n* = 26 Age 69 (13) Sex 4 M	Exp: secondary prevention education + general lifestyle counselling using motivation interviewing Con: usual care, no additional support or information given unless requested by the patient	Exp: not reported Face-to-face and phone	Exp: 3 sessions over 6 wk. Con: usual care	Self-reported exercise frequency (n of 20-min sessions/wk)
Joubert et al. [30] (2009)	Time since stroke or TIA unknown Exp: *n* = 91 Age 63 (14) Sex 53 M Con: *n* = 95 Age 68 (13) Sex 49 M	Exp: secondary prevention education + general lifestyle counselling (protocoled according to ICARUSS model), Con: usual care	Exp: general practitioner, researcher Face-to-face and phone	Exp: frequency not specified over 12 mth Con: usual care	Self-reported exercise frequency (n of ‘deliberate’ walks/wk)
Adie et al. [31] (2010)	Time since stroke or TIA < 1 months at recruitment Exp: *n* = 29 Age 73 (54–90) Sex NR Con: *n* = 27 Age 73 (54–90) Sex NR	Exp: usual care + secondary prevention education + lifestyle counselling using the TFU method, including motivational interviewing Provided by: Con: usual care	Exp: not reported Phone	Exp: at 7–10 days, 1, 2 and 4 months; 4 over 4 mth Con: usual care	Self-reported exercise min/wk
Fleming et al. [32] (2013)	Time since stroke or TIA unknown Exp: *n* = 20 Age 70 (13) Sex 10 M Con: *n* = 21 Age 71 (9) Sex 14 M	Exp: secondary prevention education + lifestyle counselling using motivational interviewing + secondary prevention education to primary care physician Con: usual care	Exp: nurse with assistance of a research physician and an exercise physiologist Face-to-face	Exp: at week 6 and after 1–3–6-9-12 mth Con: usual care	% participants self-reported to be exercising n participants deemed to be physically active (criteria not reported)
Allen et al. [33] (2009)	Time since stroke unknown Exp: *n* = 190 Age 68 (1) Sex 91 M Con: *n* = 21 Age 69 (1) Sex 99 M	Exp: secondary prevention education + general lifestyle counselling + ad hoc multidisciplinary support Con: usual care + Primary care physician is informed about individuals risk factors	Exp: Nurse Face to face, phone Con: Nurse Written material	Exp: frequency not specified over 6 mth Con: usual care	% participants self-reported to be exercising
Faulkner et al. [34] (2015)	Time since stroke or TIA unknown Exp: *n* = 29 Age 65 (11) sSex 15 M Con: *n* = 29 Age 68 (10) Sex 14 M	Exp: usual care + secondary prevention education, including group discussion using health belief model for behaviour change + supervised exercise Con: usual care	Exp: health and exercise practitioners Face to face, written material	Exp: 2 90-min exercise sessions and 1 30-min education session/wk., over 8 wk. Con: usual care	International Physical Activity Questionnaire (IPAQ) min/wk
Olaiya et al. [35] (2017)	Time since stroke or TIA unknown Exp: *n* = 283 Age median 69 (Q1:61, Q2:78) Sex 187 M Con: *n* = 280 Age median 71 (Q1:71, Q2:79) (10) Sex 176 M	Exp: secondary prevention education + general lifestyle counselling, including a management plan for the primary care physician. Con: usual care	Exp: General practitioner, nurse Face to face	Exp: frequency not specified over 6 mth Con: usual care	n participants self-reported as being physically active (≥30 min of moderate intensity activity or ≥ 20 min of vigorous intensity physical activity ≥3 times/week)
Askim et al. [36] (2018)	Time since stroke, d mean (SD): Exp: 111.3 (24.5), Con: 112.0 (17.2) Exp: *n* = 186 Age 72 (12) Sex 104 M Con: *n* = 194 Age 72 (11) Sex 127 M	Exp: physical activity coaching including goal setting + ad hoc supervised exercise Con: usual care	Exp: physiotherapist Face to face	Exp: once a mth for 18 mth	International Physical Activity Questionnaire (IPAQ) min/wk
Cheng et al. [37] (2018)	Time since stroke: < 90 days at inclusion Exp: *n* = 204 Age 57 (7) Sex 128 M Con: *n* = 200 Age 58 (7) Sex 116 M	Exp: secondary prevention education + general self-management counselling Con: usual care	Exp: nurse practitioners or physician assistants Face to face, phone	Exp: 3 group sessions and 3 individual sessions over 10 mth Con: usual care	n participants self-reported as exercising ≥3 day/wk.
Teuschl et al. [38] (2017)	Time since stroke < 3 months at recruitment Exp: *n* = 80 Age 63 (8) Sex 59 M Con: *n* = 87 Age 61 (10) Sex 63 M	Exp: cognitive training + secondary prevention education + general self-management and motivation counselling Con: usual care + advice on medical adherence	Exp: nutritionists, physiotherapists, occupational therapists, and neurologists Face to face, phone Con: phone	Exp: 45 group session over 24 months Con: usual care + 24 moths	% participants self-reported as more than 150 min moderate intensity or 75 min vigorous-intensity pa/week

*M* male, *MVPA* moderate to vigorous intensity physical activity

All 11 interventions included a form of education, motivation and/or guidance to support the participants in changing their lifestyle. Regular supervised exercise was included in 2 of the trials that specifically targeted improving physical activity [28, 34], and on an ad hoc basis in the third [36]. In 3 of the included trials a physiotherapist was involved in the intervention [28, 36, 38]. In the other 7 trials the intervention was delivered by either a case manager, a general health care professional, a general practitioner, a nurse, an exercise practitioner, or it was not stated.

The type of outcome measures used to determine the level of physical activity varied. Only one trial used an objective outcome measure to measure steps and minutes spent in low, moderate and high intensity activity time per day [28]. The other 10 trials (91%) used self-reported outcome measures [29–38]. Two trials used a standardized, validated questionnaire [34, 36], and 8 trials used general non-validated questionnaires [29–33, 35, 37, 38].

### Methodological quality

The quality assessment of the included trials is reported in Table 2, ‘PEDro scores’. Initial agreement among the 2 authors was 95% with full consensus reached through discussion. The PEDro scores ranged from 4 to 8 points (Table 2, ‘PEDro scores’). No study achieved a full score of 10 points due to lack of blinding of the participants (question 5, supplementary Table S1, PEDro scale) and the professionals responsible for the treatment (question 6, supplementary Table S1, PEDro scale), which is not possible in these types of interventions. Eight studies had a score of 6 or higher and were therefore considered to be of high quality.

**Table 2:** PEDro scores

Study	Eligibility criteria specified^a^	Random allocation	Concealed allocation	Groups similar at baseline	Participant blinding	Therapist blinding	Assessor blinding	< 15% dropouts	Intention-to-treat analysis	Between-group difference reported	Point estimate and variability reported	Total (0 to 10)
Kono et al. [28] (2013)	Y	Y	Y	Y	N	N	Y	Y	Y	Y	Y	8
Gillham et al. [29] (2010)	N	Y	N	N	N	N	N	Y	N	Y	Y	4
Joubert et al. [30] (2009)	Y	Y	Y	N	N	N	N	N	N	Y	Y	4
Adie et al. [31] (2010)	Y	Y	Y	Y	N	N	N	Y	N	Y	Y	6
Fleming et al. [32] (2013)	Y	Y	N	Y	N	N	N	Y	N	Y	Y	5
Allen et al. [33] (2009)	Y	Y	Y	Y	N	N	Y	Y	Y	Y	Y	8
Faulkner et al. [34] (2015)	Y	Y	Y	Y	N	N	Y	Y	N	Y	Y	7
Olaiya et al. [35] (2017)	Y	Y	Y	Y	N	N	Y	Y	Y	Y	Y	8
Askim et al. [36] (2018)	Y	Y	Y	Y	N	N	Y	N	Y	Y	Y	7
Cheng et al. [37] (2018)	Y	Y	Y	Y	N	N	Y	N	Y	Y	Y	7
Teuschl et al. [38] (2017)	Y	Y	Y	Y	N	N	Y	Y	Y	Y	Y	8

^a^excluded from total score

### Results of individual trials

Five out of the 11 trials found significant differences in the level of physical activity in favour of the intervention [28–30, 32, 36]. The effect size of the intervention could be determined by calculating the SMD (see Table 3, ‘Results individual studies’) in three trials only [28–30], and this ranged from 0.29 to 0.98.

**Table 3:** Results individual studies

Study	Outcome	Groups				Difference within groups		Difference between groups	Standardised mean difference
		Baseline		End intervention		End intervention minus Baseline		End intervention	
		Exp	Con	Exp	Con	Exp	Con	Exp minus Con	
Kono et al. [28] (2013)	Steps/day (accelerometer),*n*	6250 (2234)	6524 (2349)	8422 (2360)	6534 (1366)	2127 (1075 to 3268)	10 (− 907 to 927)	1888 (968 to 2808)	0.98 (0.48 to1.48)
	Time in moderate intensity physical activity, *min/day*	23 (17)	23 (20)	32 (17)	20 (15)	8 (0.2 to 126.4)	−3 (− 11.1 to 5.7)	15 (7.7 to 22.9)	0.73 (0.25 to 1.22)
Gillham et al. [29] (2010)	Self-reported exercise frequency, *n of 20-min sessions/wk*	1.2 (1.8)	1.4 (2.4)	2.6 (2.0)	1.9 (2.8)	1.4 (0.34 to 2.46)	0.5 (− 0.95 to 1.95)	0.7 (− 0.66 to 2.06)	0.29* (− 0.26 to 0.83)
Joubert et al. [30] (2009)	Self-reported exercise frequency, *n of ‘deliberate’ walks/wk*	3.9 (2.9)	4.3 (2.8)	4.7 (2.5)	3.6 (2.7)	0.8 (0.01 to 1.59)	−0.7 (− 1.49 to 0.09)	1.1 (0.35 to 1.85)	0.42 (0.13 to 0.71)
Adie et al. [31] (2010)	Self-reported exercise, *min/wk, (median [IQR])*	210 (350)	210 (300)	210,225)	150 (340)	median diff 0	median diff −60	median diff 60 *p* = 0.14	
Flemming et al. [32] (2013)	participants self-reported to be exercising, *%*	NR	NR	83%	33%				
	participants deemed to be physically active (criteria not reported), *n (%)*	6 (33)	11 (61)	3 (17)	12 (66)	−15% (− 39.0 to 11.1) NNT = − 7	−4.9% (− 23.5 to 31.9) NNT = 21	42% (− 63.0 to − 12.7) NNT = − 2	
Allen et al. [33] (2009)	participants self-reported to be exercising, *n (%)*	NR	NR	81	71			10 (−0.1 to 20)	
Faulkner et al. [34] (2015)^#^	IPAQ, *min/wk walking*	NR	NR	410 (463)	366 (430)			44 (− 191.1 to 279.1)	
	IPAQ, *min/wk moderate intensity activity*	NR	NR	328 (376)	105 (249)			223 (55.2 to 390.8)	
	IPAQ, *min/wk vigorous intensity activity*	NR	NR	494 (631)	127 (587)			367 (46.4 to 687.6)	
Olaiya et al. [35] (2017)^^^	participants self-reported as being physically active (≥30 min of moderate intensity activity or ≥ 20 min of vigorous intensity physical activity ≥3 times/week), *n (%)*	33 (11.7)	37 (13.2)	30 (11.2)	28 (10.5)	0.4% (−5.00 to 5.79) NNT = 235	2.7% (− 2.81 to 8.15) NNT = 37	0.7% (−4.65 to 6.08) NNT = 141	
Askim et al. [36] (2018)^**^	IPAQ, *min/wk walking (median [Q1, Q3])*	NR	NR	693 (198, 1386)	643 (198, 1386)			median diff 50 *p* = 0.55	
	IPAQ, *min/wk moderate intensity activity (median [Q1, Q3])*	NR	NR	240 (0, 720)	240 (0, 1350)			median diff 0 *p* = 0.55	
	IPAQ, *min/wk vigorous intensity activity (median [Q1, Q3])*	NR	NR	0 (0, 1020)	0 (0, 240)			median diff 0 *p* = 0.03	
Cheng et al. [37] (2018)	participants self-reported as exercising ≥3 day/wk., *n (%)*	153 (75)	140 (70)	135 (79)	125 (77)	−8.8% (17.5 to −0.02) NNT = − 11	−7.5% (− 16.6 to 1.76) NNT = −13	3.7% (−5.63 to 12.90) NNT = 27	
Teuschl et al. [38] (2017)	% participants self-reported as more than 150 min moderate intensity or 75 min vigorous-intensity pa/week	NR	NR	NR	NR	+ 1.3% (NR, *p* = 1.000)	+ 0.0% (NR, *p* = 1.000)	1.3% (NR, *p* = 0.862)	

Mean (SD) of groups, mean (SD) or n (%) difference within groups, mean difference (95% CI) or absolute risk reduction (95% CI, number needed to treat) between groups and standardised mean difference (95% CI)

*IPAQ* International Physical Activity Questionnaire

* In study the confidence interval of the effect calculation does not correspond to the articles conclusion that there was a significant between group difference [29]. After contacting the author it was decided to follow the study’s conclusion. This study was not of high quality (PEDRO score: 4), and therefore not included in the best evidence syntheses

^#^ Week 8 post-intervention values reported

^ 12 mth post-intervention values reported

**18 mth post-intervention values reported

As described above some of the trials specifically targeted improving physical activity levels and included either a standard or ad hoc supervised exercise component. Subgroup analyses of these 3 trials that included specific physical activity coaching and/or supervised exercise [28, 34, 36], showed that 2 trials found a significant difference in the levels of physical activity in favour of the intervention [28, 36]. For one of these trials the effect sizes of the intervention could be determined by calculating the SMD (see Table 3, ‘Results individual studies’), which were 0.73 and 0.98 [28].

### Best evidence synthesis

Based on PEDRO scores, 8 trials overall were considered to be of high quality and were included in the best evidence syntheses [28, 31, 33–38]. Two of these trials (25%) found a significant difference in favour of the intervention [28, 36], and 6 found (75%) no significant between group difference [31, 33–35, 37, 38], therefore the domain of consistent findings (≥75%, see methods) is met. The domain of precise data (see methods) is not met because in 38% of the trials the sample size was equal or below 35 for each treatment arm. Overall, this means there is moderate-quality evidence that lifestyle interventions do not lead to significant improvements in the level of physical activity in people with stroke or TIA, compared to usual care.

A subgroup best evidence synthesis including only trials with interventions that specifically targeted physical activity shows low quality evidence that such interventions are effective to improve the level of physical activity in people with stroke or TIA, compared to usual care. This is based on three high quality trials, of which two (67%) found a significant difference in favour of the intervention [28, 36]. One trial (33%) found no significant between group difference [34], therefore the domain of consistent findings (≥75%, see methods) is not met. The domain of precise data (see methods) is not met because in 67% of the trials the sample size was equal or below *n* = 35 for each treatment arm.

When only general lifestyle interventions were included in a best evidence syntheses there was high quality evidence they do not lead to significant improvements in the level of physical activity in people with stroke or TIA, compared to usual care. Of the five high-quality trials included in this this analysis, all (100%) show no significant between group difference [31, 33, 35, 37, 38]. This means that the domain of consistent findings (≥75%, see methods) is met.

## Discussion

This review found low-quality evidence that lifestyle interventions overall do not lead to significant improvements in the level of physical activity in people with stroke or TIA, compared to usual care, with only 2 (25%) of the 8 high-quality trials demonstrating positive findings. The results of the subgroup analyses suggest that only lifestyle interventions that include specific strategies targeting physical activity have a positive effect on the levels of physical activity. However, sample sizes were small, and in the majority of trials the levels of physical activity was a secondary outcome measure. Therefore, it is possible that some of the included trials were insufficiently powered to determine the effectiveness of the interventions on physical activity.

Counselling, advice, education, support and encouragement were commonly incorporated into the interventions, however descriptions were sparse. In those trials that included general lifestyle counselling, details about the relative emphasis on physical activity was not provided. Therefore, there is limited information to guide clinical practice regarding lifestyle counselling or physical activity coaching to improve physical activity levels of people with stroke or TIA.

There were more consistent findings of benefit for trials that included specific physical activity coaching and/or supervised exercise. The 2 high quality trials with significant positive findings included an exercise program as a standard part of their intervention or on an ad hoc basis [28, 36]. However, one high quality trial that included an exercise program found no significant between group differences [34]. This study had a sample size of 29 participants per arm (compared to 35 and 186 in the other two), so might have been underpowered [34]. This suggests that including an exercise program in the lifestyle intervention may lead to better results. In 2 of the 3 high quality trials that specifically targeted improving physical activity [28, 36], a physiotherapist was involved in the intervention and both had positive findings [28, 36]. Since a specific focus on physical activity and/or adding an exercise component to a lifestyle intervention might be beneficial, the involvement of experts in physical activity and exercise, such as physiotherapists may be a critical component for success.

The outcome measures used across the included trials were too diverse to conduct meta-analyses in this review. This corresponds to the conclusions of earlier reviews [16–18]. All but one study included self-reported physical activity outcome measures. Additionally, several trials measured one aspect of physical activity (e.g. taking exercise walks or participating in exercise sessions), instead of all possible types of physical activity combined. These factors may have influenced the effect estimation. Without an overall, objective measure of physical activity definitive conclusions cannot be drawn. Further high-quality research, using objective outcome measures, is needed. Our results on physical activity are in line with the recently updated Cochrane review on educational and behavioural interventions effects on physiological risk factors of recurrent stroke (e.g. blood pressure), which concluded these interventions did not lead to improvements in physiological risk factors [39].

### Limitations

A meta-analysis was not possible and, though a best evidence synthesis was conducted, the limitations to sample sizes and the use of non-objective outcome measures still call for caution when interpreting the results. A systematic review on the use of different self-reported outcome measures of physical activity concluded that measurement properties were insufficiently addressed, specifically content validity [40]. Furthermore, the follow-up period was less than 2 months in 5 of the 8 high quality trials which limits the determination of sustainability of the effects.

The search strategy used was thorough and included three of the most commonly used databases. Though, it is always possible that due to the build of the search string, not including other databases and the exclusion of papers not published in English or Dutch, trials on the subject may not have been identified. We also acknowledge that since the search was conducted it is possible that additional trials have been published on the subject. Though a search in one database (Pubmed) in March 2020 did not reveal new studies.

All studies included in this review were conducted in high income countries [28–38]. However, the World Health Organisation concludes that the middle and low-income countries have the highest incidence and death rates for stroke [1]. Further trials are needed to determine the effectiveness of lifestyle interventions in middle and low-income countries.

### Implications and recommendations for future research

Current clinical guidelines emphasise the importance of increasing physical activity levels as part of (secondary) stroke prevention [12–15]. Clinicians therefore need clear guidance on the best way to improve physical activity levels for their patients. Although a positive trend is seen for trials that include specific physical activity coaching and/or supervised exercise programs, there is currently insufficient evidence to support definitive recommendations. There is also a lack of specific detail on the content and behaviour change techniques used in these interventions which further limits implementation. In light of the fact that sustainable behaviour change has been proven very difficult both in research and clinical practice, this information is crucial [41, 42]. Recommendations for further research include better description of the content of the intervention in particular the behaviour change techniques used, more homogeneous objective outcome measures, adequate sample sizes, and longer follow-up periods [43]. Populations from middle and low income countries should also be included*.*

## Conclusion

The results of this review demonstrate high-quality evidence that general lifestyle interventions seem insufficient to improve the levels of physical activity in people with stroke or TIA. The subgroup analyses indicate that lifestyle interventions specifically targeting the levels of physical activity might be effective. Further research is needed to determine the effectiveness of combining lifestyle interventions that include behaviour change strategies specifically focusing on improving physical activity and/or supervised exercise programs to sustainably improve physical activity after stroke.

## Supplementary information

**Additional file 1.** Appendix I Search strategy.

**Additional file 2: Table S1.** PEDro scale.

## Data Availability

The current study was a systematic review and all data used was retrieved from the published articles of the included studies and their supplementary material. Therefore, all data is available from those published papers and supplementary material.

## Abbreviations

CINAHL: Cumulative Index for Nursing and Allied Health Literature
GRADE: Grading of Recommendations, Assessment, Development and Evaluations
PEDro: Physiotherapy Evidence Database
PRISMA: Preferred Reporting Items for Systematic Reviews and Meta-analyses
PROSPERO: International Prospective Register of Systematic Reviews
RCT: Randomised clinical trial
SMD: Standardized mean difference
TIA: Transient Ischemic Attack
