# Supplementary material for: General lifestyle interventions on their own seem insufficient to improve the level of physical activity after stroke or TIA: a systematic review
Source: BMC Neurol. 2020 May 1;20:168. doi: 10.1186/s12883-020-01730-3 (PMC7195782; doi:10.1186/s12883-020-01730-3)
Supplement: Supplementary file 1 — Additional file 1. Appendix I Search strategy. [file 12883_2020_1730_MOESM1_ESM.docx]

Appendix I Search strategy.

Databases: Pubmed, Embase and CINAHL

| **#1** | Search ("Stroke"[Mesh]) OR "Brain Infarction"[Mesh] OR “Stroke”[Title/Abstract] OR “Cerebro Vascular Accident”[Title/Abstract] OR CVA”[Title/Abstract] OR “Brain infarction”[Title/Abstract] OR “Cerebral apoplexy” OR Poststroke*[Title/Abstract]) |
| --- | --- |
| **#2** | Search ((("Secondary Prevention"[Mesh]) OR "Risk Reduction Behavior"[Mesh])) OR (((("Secondary prevention"[Title/Abstract]) OR "Lifestyle interventions"[Title/Abstract]) OR "Behavioural interventions"[Title/Abstract]) OR "Lifestyle modification"[Title/Abstract]) |
| **#3** | Search #1 AND #2 |
| **#4** | Search ((((("Motor Activity"[Mesh]) OR "Exercise"[Mesh]) OR "Walking"[Mesh]) OR "Physical Fitness"[Mesh])) OR (((("Motor Activity"[Title/Abstract]) OR "Exercise"[Title/Abstract]) OR "Walking"[Title/Abstract]) OR "Physical Fitness"[Title/Abstract]) |
| **#5** | Search #3 AND #4 |
| **#6** | Search ("Risk Reduction Behavior"[Mesh] OR "Life Style"[Mesh]) OR "Lifestyle intervention*"[Title/Abstract) OR "Life style intervention*"[Title/Abstract] OR "Behaviour* intervention*"[Title/Abstract] OR "Lifestyle modification*"[Title/Abstract] OR "Life style modification*"[Title/Abstract]) |
| **#7** | Search #1 AND #6 |
| **#8** | Search #5 AND #7 |
