# Supplementary material for: General lifestyle interventions on their own seem insufficient to improve the level of physical activity after stroke or TIA: a systematic review
Source: BMC Neurol. 2020 May 1;20:168. doi: 10.1186/s12883-020-01730-3 (PMC7195782; doi:10.1186/s12883-020-01730-3)
Supplement: Supplementary file 2 — Additional file 2: Table S1. PEDro scale. [file 12883_2020_1730_MOESM2_ESM.docx]

**Table S1. PEDro scale**.

| 1. eligibility criteria were specified | Yes | No |
| --- | --- | --- |
| 2. subjects were randomly allocated to groups (in a crossover study, subjects  were randomly allocated an order in which treatments were received) | Yes | No |
| 3. allocation was concealed | Yes | No |
| 4. the groups were similar at baseline regarding the most important prognostic  indicators | Yes | No |
| 5. there was blinding of all subjects | Yes | No |
| 6. there was blinding of all therapists who administered the therapy | Yes | No |
| 7. there was blinding of all assessors who measured at least one key outcome | Yes | No |
| 8. measures of at least one key outcome were obtained from more than 85%  of the subjects initially allocated to groups | Yes | No |
| 9. all subjects for whom outcome measures were available received the  treatment or control condition as allocated or, where this was not the case,  data for at least one key outcome was analysed by “intention to treat” | Yes | No |
| 10. the results of between-group statistical comparisons are reported for at least one key outcome | Yes | No |
| 11. the study provides both point measures and measures of variability for at  least one key outcome | Yes | No |
